# Synthesis and Antibacterial Evaluation of New Thione Substituted 1,2,4-Triazole Schiff Bases as Novel Antimicrobial Agents

**Published:** 2015

**Authors:** Karim Akbari Dilmaghani, Fazel Nasuhi Pur, Mahnaz Hatami Nezhad

**Affiliations:** a *Department of Chemistry, Faculty of Science, Urmia University, Urmia, Iran.*; b *Health Technology Incubator Center, Urmia University of Medical Science, Urmia, Iran.*

**Keywords:** Schiff base, *In-vitro*, *Acinetobacter calcoaceticus*, Antimicrobial agents

## Abstract

The condensation reaction of 5-(4-aminophenyl)-4-phenyl-1,2,4-triazole-3-thione with salicylaldehyde, 4-hydroxybenzaldehyde, 5-chlorosalicylaldehyde, 5-bromosalicylaldehyde, 2-nitrobenzaldehyde, 3-nitrobenzaldehyde, 4-nitrobenzaldehyde and 4-methoxybenzaldehyde in methanol results in series of new Schiff bases. The structure of Schiff bases were confirmed by ^1^H NMR, ^13^C NMR, IR and mass spectroscopy. The synthesized compounds were tested for their antimicrobial activity against bacterial (Gram negative and Gram positive) strains *in-vitro*. The synthetic compounds showed different inhibition zones against tested bacterial strains. All compounds showed significant antiproliferative activity against *Acinetobacter calcoaceticus* ATCC 23055. In detail, *Entrococcus faecalis* (Gram positive) was resistant to all prepared compounds, whereas, *A. calcoaceticus* (Gram negative) was sensitive to all compounds especially 5c, 5d and 4. *S. aureus* (Gram positive, relatively resistant to antimicrobials) showed limited sensitivity to only 5c and 5d, and it was resistant to all other compounds and only 5c exhibited low activity against *P. aeruginosa* (Gram negative). The best results belonged to 5c that showed high activity against *A. calcoaceticus* (33 mm) as well as *S. aureus* (20 mm).

## Introduction

*Acinetobacter* (Gram-negative) is a genus of opportunistic pathogens in the proteo-bacteria group, species of which are distributed in widespread, diverse habitats. These bacteria are widely distributed in hospitals, where they pose the danger of transferring resistance to other hospital-inhabiting bacteria. They are found in soil, water, and in living organisms, where they may or may not be pathogenic. They can use a varied selection of organic materials as sources of carbon. *Acinetobacters* are very resistant to antibiotics and are difficult to differentiate between species when isolated from patients (1).

The resistance of infective bacteria to present antibiotics demands developing research assigned to the discovery of new drugs in the antibacterial drug field. A wide variety of heterocyclic systems have been utilized for designing pharmaceutically active molecules. 

The 1,2,4-triazole nucleus is found in many drug structures such as anastrozole, estazolam, ribavirin, and triazolam. In addition, these compounds show antiseptic, analgesic and anticonvulsant properties (2-6). Moreover, sulfur-containing heterocycles represent an important group of sulfur compounds that are promising for use in practical applications. Among these heterocycles, thione-substituted 1,2,4-triazole ring systems have been well studied and so far a variety of biological activities have been reported for a large number of their derivatives, such as antibacterial (7-9), antifungal (10,11), antitubercular (12), antimycobacterial (13), anticancer (14,15), urease inhibition and antioxidant (16-18), diuretic (19), hypoglycemic (20), antiproliferative (21), anti-HIV (22) and anti-inflammatory (23,24) properties.

On the other hand, Schiff bases containing azomethine group attract much interest due to their synthetic availability along with antibacterial (25-27) and antitumor (28) properties.

Therefore, it is interesting to report the synthesis of a new series of compounds in which the triazole ring has been used as carrier for the Schiff base moieties.

In our previous work (29), we reported the coupling effect of thione-substituted 1,2,4-triazole nuclei to monosaccharide units on their antiproliferative activity, whereas in the present, we report the effect of adding the imine group to the nucleus on their antimicrobial activity. 

## Experimental

IR spectra were determined on a Thermo Nicolet 610 Nexus FT-IR spectrometer in KBr disks. ^1^H and ^13^C NMR spectra were recorded on a Bruker Avance-300 spectrometer at 300 and 75 MHz, respectively, in DMSO-d_6_ using TMS as the internal standard. High resolution mass spectra were obtained with a HPLC-Q-Tof system equipped with Q-TOF micro mass spectrometer (dual ESI). Melting points were measured on Philip Harris C4954718 apparatus without calibration. Thin layer chromatography (TLC) analyses were carried out on silica gel plates. All chemicals were purchased from Merck and used as received.

Ethyl 4-(benzoylamino)benzoate 1, 4-(benzoylamino)benzoylhydrazine 2, and 4-phenyl-1-[4-(benzoylamino)benzoyl]thiosemicarbazide 3 were prepared by the previously reported methods (30-32).

*Synthesis of* *1,2,4-triazole-3-thione 4*

The corresponding compound 3 (5.63 g, 12 mmol) was refluxed in 50 mL of 4N aqueous sodium hydroxide solution for 12 h. The mixture was cooled to room tempreture and then neutralized with 4N hydrochloric acid. The yellow precipitate was filtered off and then crystallized from aqueous ethanol (18). 

*Synthesis of Schiff bases 5a–h*

The respective compound 4 (1 mmol) and benzaldehyde derivative (1 mmol) were dissolved in 10 mL of methanol, and the reaction mixture was refluxed for 8 h. After cooling, the resulting precipitate was recrystallized from methanol. 

*5-(4-aminophenyl)-4-phenyl-1,2,4-triazole-3-thione (4)*

 Yield 64%. Orange powder, mp 243–245 °C. IR spectrum, ν, cm^-1^: 3450 (NH_2_), 3360 (NH), 1273 (C=S). ^1^H NMR spectrum, δ, ppm (*J*, Hz): 5.55 (2H, s, NH_2_); 6.39 (2H, d, *J *= 8.7, H Ar); 6.90 (2H, d, *J *= 8.7, H Ar); 7.26-7.31 (2H, m, H Ar); 7.46-7.53 (3H, m, H Ar); 13.81 (1H, s, NH). ^13^C NMR spectrum, δ, ppm: 112.5; 113.5; 129.2; 129.5; 129.6; 129.7; 135.5; 151.1 (C-NH_2_); 151.7 (C=N); 168.4 (C=S). Found, *m/z*: 269.0943 [M+H]+. C_14_H_12_N_4_S. Calculated, *m/z*: 269.0861.

*5-(4-((2-hydroxybenzylidene)amino)phenyl)-4-phenyl-1,2,4-triazole-3-thione (5a)*

Yield 48%. Yellowish powder, mp 190–192 °C. IR spectrum, ν, cm^-1^: 3421 (OH, NH), 1618 (CH=N), 1281 (C=S). ^1^H NMR spectrum, δ, ppm (*J*, Hz): 6.93-6.98 (2H, m, H Ar); 7.35-7.41 (7H, m, H Ar); 7.49-7.52 (3H, m, H Ar); 7.62 (1H, d, J = 8.4, H Ar); 8.90 (1H, s, CH=N); 12.69 (1H, s, OH); 14.14 (1H, s, NH). ^13^C NMR spectrum, δ, ppm: 117.1; 119.7; 122.0; 124.4; 129.2; 129.8 (2C); 129.9; 130.0; 133.1; 134.2; 135.1; 150.2; 150.6 (C=N); 160.7 (CH=N); 165.0; 169.4 (C=S). Found, *m/z*: 373.1203 [M+H]+. C_21_H_16_N_4_OS. Calculated, *m/z*: 373.1123.

*5-(4-((4-hydroxybenzylidene)amino)phenyl)-4-phenyl-1,2,4-triazole-3-thione (5b)*

Yield 50%. Orange powder, mp 196–198 °C. IR spectrum, ν, cm^-1^: 3416 (OH, NH), 1503 (CH=N), 1280 (C=S). ^1^H NMR spectrum, δ, ppm (*J*, Hz): 7.02 (2H, d, J = 8.1, H Ar); 7.06 (2H, d, J = 8.1, H Ar); 7.16 (2H, d, J = 8.7, H Ar); 7.21 (1H, s, H Ar); 7.28 (2H, d, J = 8.7, H Ar); 7.44-7.53 (4H, m, H Ar); 7.79 (2H, d, J = 8.4, H Ar); 8.52 (1H, s, CH=N); 12.64 (1H, s, OH); 14.12 (1H, s, NH). ^13^C NMR spectrum, δ, ppm: 114.6; 116.3; 120.1; 122.5; 127.6; 128.3; 129.1; 131.2; 132.3; 141.1; 153.3 (C=N); 161.2 (CH=N); 162.5; 163.7; 168.4 (C=S). Found, *m/z*: 373.1205 [M+H]+. C_21_H_16_N_4_OS. Calculated, *m/z*: 373.1123.

*5-(4-((5-chloro-2-hydroxybenzylidene)amino)phenyl)-4-phenyl-1,2,4-triazole-3-thione (5c)*

 Yield 52%. Orange powder, mp 195–197 °C. IR spectrum, ν, cm^-1^: 3425 (OH, NH), 1621 (CH=N), 1277 (C=S). ^1^H NMR spectrum, δ, ppm (*J*, Hz): 6.96 (1H, d, J = 8.4, H Ar); 7.30-7.40 (6H, m, H Ar); 7.49-7.55 (4H, m, H Ar); 7.82 (1H, bs, H Ar); 8.86 (1H, s, CH=N); 12.61 (1H, s, OH); 14.14 (1H, s, NH). ^13^C NMR spectrum, δ, ppm: 110.5; 113.5; 119.6; 122.1; 129.2; 129.5; 129.7; 129.9; 130.9; 135.0; 136.4; 138.8; 150.1; 150.7 (C=N); 159.7 (CH=N); 163.3; 169.2 (C=S). Found, *m/z*: 407.0813 [M+H]+. C_21_H_15_ClN_4_OS. Calculated, *m/z*: 407.0733.

*5-(4-((5-bromo-2-hydroxybenzylidene)amino)phenyl)-4-phenyl-1,2,4-triazole-3-thione (5d).*

Yield 54%. Yellowish powder, mp 190–192 °C. IR spectrum, ν, cm^-1^: 3422 (NH), 1620 (CH=N), 1273 (C=S). ^1^H NMR spectrum, δ, ppm (*J*, Hz): 6.92 (1H, d, J = 8.7, H Ar); 7.30-7.40 (6H, m, H Ar); 7.47-7.55 (4H, m, H Ar); 7.82 (1H, bs, H Ar); 8.85 (1H, s, CH=N); 12.60 (1H, s, OH); 14.11 (1H, s, NH). ^13^C NMR spectrum, δ, ppm: 110.5; 113.5; 120.4; 122.1; 129.2; 129.5; 129.7; 129.9; 130.9; 135.0; 136.4; 138.9; 150.1; 150.7 (C=N); 160.0 (CH=N); 163.3; 169.1 (C=S). Found, *m/z*: 451.0310 [M+H]+. C_21_H_15_BrN_4_OS. Calculated, *m/z*: 451.0228.

*5-(4-((2-nitrobenzylidene)amino)phenyl)-4-phenyl-1,2,4-triazole-3-thione (5e* **)**

Yield 46%. Orange powder, mp 198–200 °C. IR spectrum, ν, cm^-1^: 3432 (NH), 1623 (CH=N), 1275 (C=S). ^1^H NMR spectrum, δ, ppm (*J*, Hz): 7.20 (2H, d, J = 8.1, H Ar); 7.34-7.40 (4H, m, H Ar); 7.49-7.52 (3H, m, H Ar); 7.77 (1H, t, J = 7.5, H Ar); 7.85 (1H, t, J = 7.5, H Ar); 8.09-8.12 (2H, m, H Ar); 8.81 (1H, s, CH=N); 14.14 (1H, s, NH). ^13^C NMR spectrum, δ, ppm: 113.8; 121.8; 123.4; 124.2; 126.4; 128.2 (2C); 128.9; 131.0; 135.1; 135.2; 137.7; 148.6; 151.6; 152.9 (C=N); 160.8 (CH=N); 168.1 (C=S). Found, *m/z*: 402.1107 [M+H]+. C_21_H_15_N_5_O_2_S. Calculated, *m/z*: 402.1025.

*5-(4-((3-nitrobenzylidene)amino)phenyl)-4-phenyl-1,2,4-triazole-3-thione (5f* **)**

Yield 52%. Orange powder, mp 196–198 °C. IR spectrum, ν, cm^-1^: 3428 (NH), 1626 (CH=N), 1277 (C=S). ^1^H NMR spectrum, δ, ppm (*J*, Hz): 7.26 (2H, d, J = 8.7, H Ar); 7.34-7.40 (4H, m, H Ar); 7.47-7.54 (3H, m, H Ar); 7.80 (1H, t, J = 8.1, H Ar); 8.31-8.38 (2H, m, H Ar); 8.69 (1H, bs, H Ar); 8.76 (1H, s, CH=N); 14.15 (1H, s, NH). ^13^C NMR spectrum, δ, ppm: 113.5; 121.8; 123.4; 124.2; 126.4; 129.2; 129.7; 129.8; 129.9; 131.0; 135.2; 137.7; 148.6; 150.7; 152.5 (C=N); 160.8 (CH=N); 169.1 (C=S). Found, *m/z*: 402.1105 [M+H]+. C_21_H_15_N_5_O_2_S. Calculated, *m/z*: 402.1025.

*5-(4-((4-nitrobenzylidene)amino)phenyl)-4-phenyl-1,2,4-triazole-3-thione (5g)*

Yield 45%. Yellowish powder, mp 194–196 °C. IR spectrum, ν, cm^-1^: 3409 (NH), 1625 (CH=N), 1280 (C=S). ^1^H NMR spectrum, δ, ppm (*J*, Hz): 7.27 (2H, d, J = 8.7, H Ar); 7.34-7.40 (4H, m, H Ar); 7.48-7.54 (3H, m, H Ar); 8.15 (2H, d, J = 8.7, H Ar); 8.35 (2H, d, J = 8.7, H Ar); 8.76 (1H, s, CH=N); 14.10 (1H, s, NH). ^13^C NMR spectrum, δ, ppm: 121.8; 124.4; 124.5; 129.2; 129.7; 129.8; 129.9; 130.3; 135.1; 141.6; 149.5; 150.7; 152.5 (C=N); 160.9 (CH=N); 169.1 (C=S). Found, *m/z*: 402.1105 [M+H]+. C_21_H_15_N_5_O_2_S. Calculated, *m/z*: 402.1025.

*5-(4-((4-methoxybenzylidene)amino)phenyl)-4-phenyl-1,2,4-triazole-3-thione (5h)*

Yield 42%. Yellowish powder, mp 180–182 °C. IR spectrum, ν, cm^-1^: 3426 (NH), 1627 (CH=N), 1247 (C=S). ^1^H NMR spectrum, δ, ppm (*J*, Hz): 3.82 (3H, s, OMe); 6.99 (2H, d, J = 8.7, H Ar); 7.04 (2H, d, J = 8.7, H Ar); 7.14 (2H, d, J = 8.7, H Ar); 7.20 (1H, s, H Ar); 7.28 (2H, d, J = 8.7, H Ar); 7.43-7.54 (4H, m, H Ar); 7.84 (2H, d, J = 8.7, H Ar); 8.46 (1H, s, CH=N); 14.09 (1H, s, NH). ^13^C NMR spectrum, δ, ppm: 55.9 (OCH_3_); 113.7; 114.8; 119.9; 122.3; 127.8; 128.3; 129.2; 131.2; 132.3; 140.3; 153.8 (C=N); 161.2 (CH=N); 162.6; 163.9; 168.2 (C=S). Found, *m/z*: 387.1362 [M+H]+. C_22_H_18_N_4_OS. Calculated, *m/z*: 387.1280.

*Bacterial strains*

The antibacterial activity of the compounds was tested against Gram positive and Gram negative bacterial strains including *Enterococcus faecalis* ATCC 29212 and *Staphylococcus aureus* ATCC 25923, as well as Gram negative strains covering *Acinetobacter calcoaceticus* ATCC 23055, *Escherichia* *coli* ATCC 25922 and *Pseudomonas aeruginosa* ATCC 27853. 

*Preparation of test compounds and anti-bacterial activity assays*

The antibacterial activity of compounds was assayed with the method of Parekh et al. (33) with some modifications. In brief, solutions with 10 µg/µL concentrations of each compounds in DMSO (Merck) has been prepared. A loop full of defined strain was inoculated in 25 mL of Nutrient Broth medium (BBL) and was incubated for 24 h in 37 °C. Mueller Hinton Agar (MHA) (Merck) plates were prepared according to the manufacturer's recommendations by dissolving 34 g of the medium in 1000 mL of distilled water. 30 mL of autoclaved media were added into a 10 cm plate. Inoculation of each strain was done by the pour-plate method. 200 μL of the activated strain was added into the MHA medium in 45 °C and after proper homogenization were distributed into a Petri-dish. The complete microbiological procedures were performed in a laminar airflow to maintain aseptic conditions. After solidification of the media, a well was made in the in the MHA with a sterile glass tube (6 mm) and 50 μL of drug compound was added into the well. 50 μL of DMSO was inoculated into another well as negative control. The antibacterial activities of drug compounds were determined by measuring the inhibition zone formed around each well against defined bacterial strain. Erythromycin and Cephalothin were used as standard drugs for antibacterial effects against Gram positive bacteria, Ampicillin and Trimethoprim/sulfamethoxazole used as standard for Gram negative bacteria. All strains were resistant to DMSO (negative control).

## Results and Discussion

**Figure 1:**
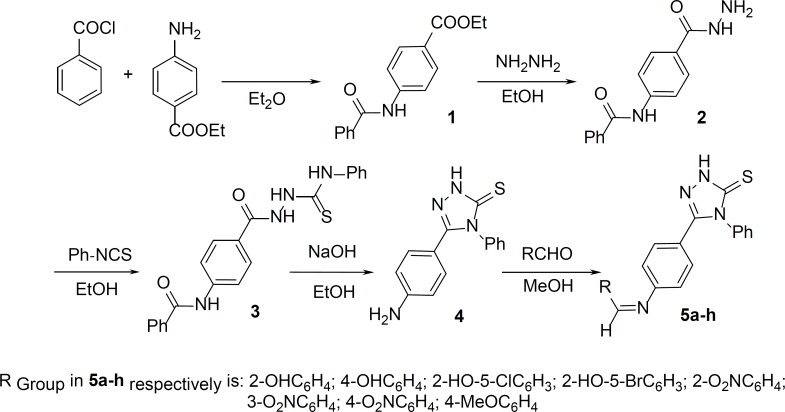
General synthetic pathway for the synthesis of triazole Schiff bases.

5-(4-aminophenyl)-4-phenyl-1,2,4-triazole-3-thione and its Schiff base derivatives were obtained by a versatile and efficient synthetic route outlined in the Figure 1. The reaction of benzoyl chloride and benzocaine in diethyl ether gave ethyl 4-(benzoylamino)benzoate 1. The hydrazide 2 was prepared by refluxing compound 1 with hydrazine hydrate. The resulting compound was treated with phenyl isothiocyanate providing 4-phenyl-1-[4-(benzoylamino)benzoyl]thiosemicarbazide 3, which was cyclized in sodium hydroxide into 5-(4-aminophenyl)-4-phenyl-1,2,4-triazole-3-thione 4. Schiff bases 5a–h, were synthesized by the reaction of the corresponding 1,2,4-triazole-3-thione 4 and benzaldehyde derivatives in MeOH.

The structure of the Schiff bases were confirmed by appropriate spectroscopic methods such as ^1^H NMR, ^13^C NMR, IR and high resolution mass spectroscopy (HRMS). The chemical shifts of imine (CH=N) and N–H protons were observed as singlets at 8.45–8.90 and 14.08–14.15 ppm, respectively, in ^1^H NMR spectra. Hydroxyl protons of the derivatives 5a–d showed resonance within the range of 12.60–12.70 ppm as a result of intra-molecular hydrogen bond between the hydroxyl proton and the imine group nitrogen. The chemical shifts of carbon of imine group and C=S group showed resonance within the range of 159.7–161.2 and 168.1-169.4 ppm, respectively, in the ^13^C NMR spectra. The imine and C=S group stretching vibration in the Schiff bases 5 was indicated by 1618–1627 and 1247-1281 cm^–1^ band, respectively, in the IR spectra. 

The *in-vitro* antibacterial activity of the synthesized compounds in DMSO against some of the most important Gram positive and Gram negative infectious agents were shown in Table 1.

**Table 1:** *In* *-* *vitro *Antibacterial activity of the synthesized compounds against bacterial strains (Concentration = 10 µg/µL)

**Inhibition zone (mm) each well contains 50 µL of the solution**															
Synthesized compounds	**4**	**5a**	**5b**	**5c**	**5d**	**5e**	**5f**	**5g**	**5h**	**Erythromycin** **(5 µg)**	**Cephalothin** **(30 µg)**	**Ampicillin** **(10 µg)**	**Trimethoprim** **/sulfamethoxazole** **(1.25/23.75-µg)**	**Ciprofloxacin** **(5 µg)**	**Imipenem** **(10 µg)**
Bacterial strains															
***Acinetobacter calcoaceticus *** **ATCC 23055**	28mm	20mm	20mm	33mm	30mm	14mm	19mm	10mm	15mm	Nt	Nt	18 mm	25mm	Nt	Nt
***Staphylococcus aureus *** **ATCC 25923**	R	R	R	20mm	15mm	R	R	R	R	20mm	27 mm	11 mm	22mm	Nt	Nt
***Escherichia coli ATCC 25922***	R	R	R	12mm	12mm	R	R	R	R	Nt	20 mm	14 mm	24mm	Nt	Nt
***pseudomonas aeruginosa ATCC 27853***	R	R	R	14mm	R	R	R	R	R	Nt	Nt	Nt	Nt	25 mm	31 mm

The synthetic compounds showed different inhibition zones against tested bacterial strains. *Entrococcus faecalis* (Gram positive) was resistant (“R”) to all prepared compounds, whereas *Acinetobacter calcoaceticus* (Gram negative) was sensitive to all compounds especially 5c and 5d. As shown in the table, these compounds showed high antibacterial effects with respect to different kinds of antibiotics such as Ampicillin and Trimethoprim/sulfamethoxazole which are normally used for treating such infections.

The best results in the Table 1 belonged to 5c that showed high activity against *A. calcoaceticus *(33 mm) as well as *Staphylococcus aureus* (20 mm). 

Only compounds 5c and 5d exerted comparable activity to reference antimicrobial Erythromycin, Ampicillin and Trimethoprim/sulfamethoxazole against *S. aureus* (Gram-positive, relatively resistant to antimicrobials).

*E. coli *(Gram negative) showed limited sensitivity to only compound 5c and 5d and their activities in comparison to Ampicillin were just slightly weaker. Also, only 5c showed a low activity against *P. aeruginosa* (Gram negative) which was not comparable to the standard compounds of Imipenem and Ciprofloxacin (Table 1).

In general, compounds 5c and 5d showed more antimicrobial activity than the other tested compounds and only these Schiff base derivatives were more active against *A. calcoaceticus *than “parent” amine 4 (Table 1).
